# Factors affecting evidence-use in food policy-making processes in health and agriculture in Fiji

**DOI:** 10.1186/s12889-016-3944-6

**Published:** 2017-01-09

**Authors:** Gade Waqa, Colin Bell, Wendy Snowdon, Marj Moodie

**Affiliations:** 1 C-POND, Fiji School of Medicine, College of Medicine, Nursing and Health Sciences, Fiji National University, Suva, Fiji; 2 Deakin Health Economics, Centre for Population Health Research, Faculty of Health, Deakin University, Melbourne, Australia; 3 Global Obesity Centre, Centre for Population Health Research, Faculty of Health, Deakin University, Melbourne, Australia

**Keywords:** Evidence-use, Policy, Policy-making process, Organization systems

## Abstract

**Background:**

There is limited research on the use of evidence to inform policy-making in the Pacific. This study aims to identify and describe factors that facilitate or limit the use of evidence in food-related policy-making in the Health and Agriculture Ministries in Fiji.

**Methods:**

Semi-structured face-to-face interviews were conducted with selected policy-makers in two government ministries that were instrumental in the development of food-related policies in Fiji designed to prevent Non-Communicable Diseases (NCDs). Snowball sampling was used to recruit, as key informants, senior policy-makers in management positions such as national advisors and directors who were based at either the national headquarters or equivalent. Interviewees were asked about their experiences in developing food-related or other policies, barriers or facilitators encountered in the policy development and implementation process and the use of evidence. Each interview lasted approximately 45–60 minutes, and was conducted in English. Audio-recorded interviews were transcribed, thematically coded and analyzed using N-Vivo 8.0 software.

**Results:**

Thirty-one policy-makers from the Ministry of Health and Medical Services (MoHMS *n =* 18) and the Ministry of Agriculture (MoA *n =* 13) in Fiji participated in the study. Whilst evidence is sometimes used in food-related policy-making in both the Health and Agriculture Ministries (including formal evidence such as published research and informal evidence such as personal experiences and opinions), it is not yet embedded as an essential part of the process. Participants indicated that a lack of resources, poor technical support in terms of training, the absence of clear strategies for improving competent use of evidence*,* procedures regarding engagement with other stakeholders across sectors, varying support from senior managers and limited consultation across sectors were barriers to evidence use. The willingness of organizations to create a culture of using evidence was reported as a facilitator.

**Conclusion:**

The use of evidence in policy-making will only become a reality in Fiji if it is a formalized part of the government’s policy-making systems. A systems approach to food-related policy-making and implementation may achieve this by helping Ministries manage the complex and dynamic nature of food-related policy-making in Fiji.

## Background

Non-communicable Diseases (NCDs) are a growing problem and a major cause of disability and death in Fiji. The Global Burden of Disease study [1] found that ischemic heart disease, diabetes mellitus and cardiovascular disease were the highest ranking causes of years of life lost and years lived with a disability in Fiji in 2010. Fiji like other low and middle-income countries in the Pacific region is actively seeking a comprehensive set of solutions to combat the rising level of NCDs.

Food-related policy is a promising strategy for population health [2–4] as direct policy actions help create environments conducive to healthy diets. Promising policies include restrictions on the marketing of unhealthy foods and non-alcoholic beverages to children [5], nutrition labeling, and food taxes and subsidies [2]. Such policies have been widely encouraged, but there has been insufficient use overall of evidence-informed policy initiatives [2, 6]. A number of reasons are likely to hinder evidence-informed policy implementation including debate over what is considered ‘evidence’, the robustness and applicability of evidence and the availability of the evidence to policy-makers [7, 8]. Additionally, supporting research use in policymaking entails cultural, institutional and political challenges [9]. An understanding of the role of the institutional structures, the political systems that shape the capacity of governments to develop effective policies and cultural resistance that may include indifference and lack of research skills and time, are all critical [10]. Policy-makers need ready access to relevant, quality evidence but do not necessarily invest time in critical appraisal. This leads to poorly informed decision-making and ultimately less efficient and ineffective policies.

Evidence-based policy-making is characterized as a systematic approach to accessing, appraising and using evidence to shape the decision-making processes [11]. However, there are risks that evidence can be misused in policy development. Policy decisions made in health and agriculture sectors in many countries are driven by different stakeholders, including the media, funding agencies, and special interest groups [12–14]. Parkhurst [15] discussed how political interests can drive the distortion or selective inclusion of evidence, leading to invalid conclusions. An example of where this type of technical bias has included evidence creation is the tobacco’s industry’s manipulation of research findings to suggest that smoking is less harmful than is actually the case [16]. While examining the way in which policy drives the research agenda, Smith argues that rather than thinking in terms of research supplying evidence, it is more productive to look at how it generates ideas that can shape the policy-making process [17]. It is however critical to have a high level of integrity in analysing and interpreting data and support for an ‘evidence-based’ policy solution [18] when considering how evidence informs policy [8, 19].

Effective use of evidence is determined by the policy-maker’s ability to access and analyse the best available evidence, and apply it to the formulation of policies. However, there is often limited capacity and resources for supporting the development of evidence-informed policies [20–22]. Obstacles to the use of evidence in policy-making include a lack of time and the skills required to acquire evidence [8, 23, 24], the non-availability of research evidence at the time when it is required [24–26] and poor dissemination of information useful for decision-makers [27–30]. Additionally, consideration of the variable sources of evidence for complicated and complex problems in both health and agriculture sectors is essential [14]. In health and nutrition, there is often heavy reliance on medical studies and cost effectiveness analyses [31]. In contrast, the agriculture sector tends to rely more on social science research which might be seen as less rigorous in design [32].

Although the Fijian government has recognized the importance of changing the obesogenic environment through multisectoral collaborations [33], barriers related to collaboration between health and non-health sectors, and within and across sectors still exist [34–36].

Food-related policy-making and implementation needs strengthening in Fiji. However, experience suggests that achievement of this aim is complex. Firstly, work is required to help other sectors recognize the importance of reducing NCDs; for example, the trade sector needs to be involved given that much of the unhealthy food available in Fiji is imported [37, 38]. Secondly, policy enforcement is challenging. For example, in 2002 Fiji implemented a ban on mutton flap sales under a trading standard regulation not under the MoHMS. For a number of reasons however, it has not been well enforced and therefore the impact is likely to have been limited [39]. Thirdly, private sector agendas can affect policy decisions. For example, taxes on sugar-sweetened beverage have been adopted and then removed multiple times in Fiji over recent decades as a consequence of major objections and debates, despite the implementation of taxes being quite straightforward [34, 39, 40]. Thow notes that, in Fiji and other Pacific Islands, barriers to the development and implementation of effective food policies include a narrow focus solely on health concerns (not taking into account policy issues relevant to other sectors), limited engagement with other sectors in proposing and developing such cross-sectoral policies, and a lack of clear enforcement mechanisms [39]. The Fiji government, like its counterparts in many low- and middle-income countries, has limited economic and human resources, low access to technology, and inadequate access to evidence on some issues for sound decision-making, but nevertheless supports inter-sectoral collaboration during the development of policy [41, 42].

This study aims to explore what is perceived to be ‘appropriate evidence’ by the different policy-makers, and how perceptions of this differ in the health and agriculture sectors in Fiji.

## Methods

### Study design and sampling

This qualitative study entailed a collective case study design. The study builds on the Pacific Obesity Prevention In Communities (OPIC) project which took place from 2004–2009 [43] and the Translational Research on Obesity Prevention In Communities (TROPIC) project in 2010–2012 [21, 44]. Whilst the OPIC project targeted obesity amongst adolescents, a sub-study focused on food policy and identified potential policies and ranked them in terms of their likely cost-effectiveness and feasibility as measures to curb NCD growth [45]. TROPIC targeted policies to tackle obesity and NCDs in Fiji and assessed how policy-making could be increased, and whether a knowledge brokering approach (focused on increasing access to and use of evidence) would be effective [44]. It developed the skills of key staff from four government ministries and two non-government organisations in evidence-use and policy brief development. In the current study, we chose to work with the two government ministries (MoHMS and MOA) that were directly involved in the development of the food-related policies in Fiji and had previous involvement in the OPIC and TROPIC programs. We decided to work in two ministries because we recognized that organizations have their own distinct “cultures” and do not necessarily use the same language, nor have the same attitudes and expectations about the value of evidence for policy. The involvement of the two ministries may provide a better understanding of the health and agricultural policy processes, provide insights into the dynamic process that drives evidence-use in policy-making in different sectors and insight into what constitutes appropriate evidence.

Endorsement to work with the two selected government ministries was secured from their respective Permanent Secretary. Once secured, snowball sampling was used to recruit senior policy-makers from the two ministries who had relevant experience in policy development. Sampling continued until saturation of information from the interviews was reached. High-level policy-makers were defined as senior government officials with management positions such as national advisors and directors who are based either at the national headquarters in Suva or equivalent. Previous involvement in the OPIC and TROPIC projects made these participants ideal interviewees as a starting point for this study.

Potential participants were initially contacted by email, followed by a phone call detailing how she/he had been nominated. A written invitation to take part in the project was provided along with a plain language statement outlining the study purpose; the requirements of participants; and the steps that would be taken to maintain confidentiality. All participants provided verbal and written consent.

### Data collection

Semi-structured interviews of approximately 45–60 min’ duration were conducted in English in April and May 2015 by the lead investigator, generally in the interviewee’s office at a time convenient to them. An interview guide was developed using a combination of open-ended and probing questions. Interviewees were asked about their experiences in developing food-related policies or any other policies they had been involved in. This included questions about barriers encountered in the development and implementation process and the use of evidence in that process. Each interview was audio recorded using a digital recorder, with the permission of the interviewee.

Alongside the interviews, key documents related to the development of food-related policy in Fiji were reviewed to ensure a better understanding of the nature and level of evidence used in the policy development process. These included publically available documents (e.g. policy documents, government reports, speeches, news media and public submissions) and those produced for internal use (e.g. minutes of meetings, internal reports, cabinet papers, memoranda or similar correspondence) subject to availability. A formal process was followed to gain access to minutes of the high-level meetings as well as the Cabinet papers; this required completion of a Data Request Form lodged to the Data registry of the MoHMS and personal requests were made to officials of the MOA.

### Data management and analysis

Trained transcribers transcribed digital recordings of interviews. The transcribers validated all transcriptions by proofreading against the audio file and editing the transcript file accordingly. The interviewer audited all transcripts for accuracy. The recordings were de-identified to ensure confidentiality at the end of each interview and before transcribing. All transcripts were entered and managed using N-Vivo 8.0 software to identify emerging themes or patterns that related to study objectives. Key documents were also mapped against the evidence received from interviews.

The study was managed by researchers based at the Pacific Research Centre for the Prevention of Obesity and Non-communicable Diseases (C-POND), School of Medicine, Fiji National University in Suva, in collaboration with researchers at Deakin University, Australia.

## Results

### Participants

Thirty-one policy-makers from the MoHMS (*n =* 18) and the MOA (*n =* 13) in Fiji participated in the study. With approval to access key internal documents, the authors were able to access minutes of relevant meetings; analysis of these showed documentation that some policy proposals were recommended for resubmission due to insufficient evidence being used to justify policy issues. Other accessed documents such as internal reports and cabinet papers made effective use of evidence.

### Evidence

We observed variation among participants in what was understood by the term evidence and this should be kept in mind when considering how they reported using evidence.

For example, “…*evidence in agriculture is very critical and we collect more field data because agriculture is applied science.”*

### Influences on evidence-use in food related policy development

The analysis identified three broad themes that influenced the use of evidence in the development and/or the implementation of food-related policy in Fiji: access to credible evidence, collaboration across sectors to get the right technical advice, and competence in collecting, analyzing and critically appraising evidence.

(i) Access to credible evidence

The interviews revealed that MoHMS and MOA policy-makers depended on information systems, particularly Internet connected computers, to access evidence and independent advice. Participants referred to information systems as tools provided by their organizations that facilitated access and evidence-use in decision or policy-making. Overall, participants described both facilitators and barriers to accessing quality, current evidence. One participant indicated that they had computers to help them access the findings of research studies; “…… *most of the stations now have computers and have access to internet, we want to get computers to every station”.* The need for accessing library services for easy access of evidence was raised by one participant: “….*we used to have our own library; currently we don’t have a library ….”.* Another astute observation was that credible evidence was particularly hard to access “*…..the internet is always available but credible evidence is sometimes hard to access because you need to know* [23, 24]*like HINARI and PubMed, I think you need to pay to be able to access evidence from such credible websites…………..”*.A common line of response was that participants felt they did not have the time to review evidence with the rigor they would like; ”….*time is one of the major barriers…. especially on the various phases to produce this policy….needs to be done otherwise there will be high level of irrelevance in the whole policy (document)”.*

Government records were the second main source of evidence but they were often incomplete, inaccessible, or not valued for a range of reasons. One respondent said: “… *there was no policy document available to guide decisions, but there were number of studies done in the sector on production, studies on market, studies on collection center, studies on prices, studies on value added; all these studies are (kept) there in the ministry (records) but there is no document that captures all these findings. These were very comprehensive studies …conducted by the various agencies and universities but there is no document that put together these various findings into a direction that the ministry could focus …”.*

Whilst many participants believed their organizations were providing some support to facilitate the use of evidence in policy development, barriers were noted relating to a lack of proper documentation of what is actually happening in the field, the tools used for data collection and the lack of ability to effectively verify the data. One respondent said *“…we need to change the data reporting template…”* and another respondent said, *“……lack of timely information and poor planning of national surveys…”.* Evidence can be translated into a usable form and is used in policy-making but a lack of organized data can be a barrier; one respondent noted: “*we struggled to prepare the paper to justify the need to support the…….GDP of agriculture and having (proper) records to justify the cost benefit to government ….”.*

#### Impact of poor access

The impact of poor access to credible evidence and the incompleteness of government records were noted as having slowed down the policy-making process resulting in less relevant policy. One participant said: “….*the main reason of (proposed) policy not being accepted was because of little evidence used, …but again when we assess the data from the record (received), it was not that strong ……. we don’t have supply volume over the years, their records (from field officers) are not serious with no proper documentation …., we struggled to put information together to justify …”*. Many participants stated that insufficient evidence in policy proposals prolonged the process of policy-making. One respondent stated: *“…policy proposals having not enough evidence are usually sent back for reconsideration*”, whilst another said that *``…policy will be endorsed quickly if good evidence is shown to support the need”.*

#### Potential strategies to improve access

Some strategies for improving access to credible evidence along with the problems that still exist were discussed by participants. One participant said: “…*we are improving our communication and mobility ….since we don’t have data communication linkages with most of our locality officers….... The ministry is currently giving out laptops, computers, and flash nets* [broadband internet *modem* or dongle] *……, we are in the process of purchasing motorcycles ……to improve (our) reach to the farmers and get those information that we lack especially with the data from the fields…. ”.*

(ii) Collaboration across sectors to get the right technical advice

Another theme described by participants as hindering or facilitating the use of evidence in policy-making was the process of consultation and collaboration across sectors. Sectors include other government ministries, non-government organisations and civil societies with similar interests related to food policies.

#### Relationships and partnerships

Developing and working in partnerships with stakeholders was described by many participants as both a barrier and facilitator of evidence-use. One respondent said: *“…..some areas of concern are the food safety and food quality. We still believe that the role should be (shared) between the two ministries (Health and Agriculture), they should be working together with policies involving (food) production, processing and going into retailing processes, that chain should be well understood (*by both parties*)….. how food standard food quality policies can address the whole chain …”.* The importance of receiving valuable technical advice from partners across sectors and how they affect policy outcomes was also raised; “….*the Bureau of Stats is one of the important partners in policy planning as they provide us with the trend of trade data, we somehow liaise with our embassy in trying to see the demand from the overseas markets …”.* Developing a cross-cutting policy is a complex process involving multiple factors and actors. One participant said: *“… smoking, nutrition and alcohol are all trade issues …but the key players are trade and multi-national and we are struggling as how best to work with them”.*

#### Getting the right people involved

The importance of engaging and communicating with the right people during collaboration was raised in the context of addressing the problems or policy challenges. One of the respondents said: *“…the consultation effort which needs to be very much inclusive …in getting the right people so that at least they provide the right technical advice …to some extent, (the) Attorney General’s office needs to be incorporated in every consultation regarding trade.* ….…”. Staff turn-over also affects the engagement process across government ministries: “…*length of time senior staff spent in leadership level….lots of turnover in workforce….engagement and consultation process needs to be reviewed as right now is whom you know….”.*

#### Challenges of collaboration

Many participants discussed the importance of achieving a balance between the right evidence and the skills required in engaging with other players outside of their own ministry. One participant said “…..*our approach to policy is very much a medical approach and when you talk to government to create legislation, we have to move away from the medical approach. It has to be socio-economic approach and for that we need an economist or somebody who can think health economics and change our disease statistics to health economic argument ……they can understand the argument from the socio-economic perspective or environment in terms of legislation ……biggest challenge ……………….”*. Another challenge of multisectoral collaboration is that different types of evidence are seen to be important in the different sectors. One participant shared “*We are not reporting on health loss and not talking financial or economic language when dealing with trade and finance and we fail to convince them using…”.*

#### Potential strategies for improving collaboration

Weighing the benefits and seeking common ground was suggested as a good strategy for collaboration. One participant said: “……*we looked at where the problems are with advertising (*of junk food to children*), where the peak hours for all the junk foods, then with the assistance of WHO [World Health Organization] got the cabinet paper ready but the cabinet paper had been studied, endorsed and then sits with XX’s office…..for close to two years now. We are having difficulty with the other stakeholders ……..meaning the wholesalers, the producers, the commercial soft drinks, ………. they threatened that (the policy) will create unemployment, and trade doesn’t want that* …..”.

(iii) Competence in collecting, analyzing and critically appraising evidence

The third clear theme was staff competence in collecting, analyzing and critically appraising evidence.

#### Making sense of evidence

Enhancing capacity to promote greater use of evidence in policy and decision-making is crucial in any government system. One respondent noted: “…*we have our statistics unit here but we need to know how to analyze and interpret data correctly*…” Another participant commented “….*we did not do our homework well, no supportive documents ….. it went from the lower house thrown back again twice, …….we were confronted with questions in our third approach but we have lots of evidence this time to justify the case, it’s just a matter of … understanding the needs, what type of evidence is needed and where to get it from* …..”*.*

Lack of capacity and skills to systematically document field research and analyze routine data is a constraint to the use of evidence. The comment of one policy-maker illustrates this; *“……..it goes back to proper documentation; making sure that the ministry is documenting each and every process…. and is kept in a very systematic way and is accessible to people needing that information; the people that would be getting that information are also knowledgeable in how to get them like electronically …”.* Another participant said *“…. we need (more) training to conduct survey, how to analyze it, which (*statistical*) package to use ……we even need more consultation and integration within the ministry and that is something we are working towards to”.*

Some potential strategies highlighted by participants for improving competent use of evidence included the need to increase networks and research and training.

#### Increased networks to strengthen communication and exchange

Some participants felt that interaction and communication were the keys to improving competent use of evidence; one said: “………*my experience with the food safety regulation is that we have to go through the media… consult the people too for their comments…..other people they might have the expertise…”.*

The importance of networking was also discussed*: “… our network with food industries has been very good… trying to get fisheries products … to the EU so we bring in all fish factories and so another reason why we have to update our legislation not only based on CODEX but also based on EU requirements”.*

The diversity of evidence gathered from providers was also highlighted as an essential component of understanding the views of different stakeholders. For example, some farmers are constantly making adjustments in their farms for smooth operations and profitability to enhance the financial return of the farm: “…(*we also*) *document farming system to see the profitability …. monetary crop like sugar cane is easy where you have some integrated system and sometimes farmers complain about this. We know that they are making money from some other things that can only be showing if you do some farming system analysis for the whole farmer’s budget, so those are the two areas that we champion and monitor with all the stakeholders within the ministry…”.*

#### Research and training

The need for funding of scientific research was identified. One participant remarked: “*… if we need to develop policies on NCDs and Communicable disease or any other policy issues ….. need funding …. how effective is that policy in terms of achieving what you actually set up to achieve in the first place, then sometimes the policy can be outdated, you know you might develop it now because of the changes in health system, these things change, and so as finance policy”.*

Whilst the importance of training policy-makers in adopting a culture towards the use of evidence was identified, no training and educational programs have been developed. “Firstly, *at this (management) level, we are expected to think policy but we are not trained to do policy, most of us came out from operational and most of our work here still is operational ……we need to attend training for human resource development, leadership and governance in policy-making. Secondly; ….. once you think policy the next level is evidence based, there is lack of (skills to access and use) evidence or the empowerment to be able to seek relevant evidence using technology that is available to apply this…..”.* Strengthening organizational capacity requires significant investment in both financial and human resources. One participant said: “….*well right now, our Statistics unit is conducting training with our staff…..recruiting staff to cater for the needs and these are the people that are going to the field…”*.

## Discussion

This study is important and innovative because it captures policy-makers’ views and experiences on the use of evidence in food-related policy-making in both the Health and Agriculture Ministries in a middle-income country. The study findings indicate that whilst policy-makers value evidence in developing food-related policies, challenges of having a shared understanding of what evidence is, technical knowledge and tools to access and use sound and relevant evidence, and collaboration with other government sectors still exist. The low level of uptake of evidence may be attributed to these challenges and limited support for the use of evidence in decision-making within their organisation.

### Access

Insufficient access to evidence identified from the perspectives of the interviewees illustrates how their work settings are driven by the constraints in organizational support towards use of evidence in policy-making. While it is important for all policy-makers across sectors to have access to and share evidence with other stakeholders, this is often not the case, particularly if an organization’s data or information management policy does not accommodate open-access. In addition, while organizational barriers limited the impact of using evidence, the challenge for these organizations was to introduce appropriate research tools for managing and analyzing research data such as N-Vivo 8.0 software [8, 20, 21, 30]. Further, strengthening of organizational capacity and development of structures and processes that support evidence-uptake and data exchange from other sources were established to increase capacity to access research evidence [30, 46, 47]. For example, other studies have noted improvements in the use of evidence in policy-making when internet access was expanded and library resources were updated [20, 48]. Based on these findings, both the MoHMS and the MOA may benefit from investment in an open access data repository system that stores, manages, indexes, preserves and shares research outputs produced by university researchers, conference papers and summary reports of national surveys. Whilst making evidence accessible to its intended users can be expensive, self-archiving using open access has been proved successful [49, 50]. However, this cannot happen without international consensus [51]. Grey literature including reports not usually formally published, important government-planning documents, and other information for wider dissemination used in policy decisions that affect food-related policies, should also be included.

### Collaboration

One of the reasons cited for inadequate use of evidence included the general lack of formal processes for engaging and collaborating with other stakeholders. The evidence-seeking patterns of policy-makers as shared in the interviews depend on their access to cross-governmental information systems or websites and their contacts with colleagues; this created an environment where they primarily work in isolation [39, 40]. Ultimately, this situation has negative consequences; it is important to get that part of the policy-making system right in terms of providing the required support [26, 30, 52, 53]. Increasing interaction and communication between and across stakeholders was identified as key to improving the use of evidence in policy-making, both in the interviews and by others in the literature [27, 29, 30]. This is important as in the case of health and trade, some issues produced closer cooperation between the two sectors, whilst others have exposed tensions between the goals of (a) protecting health and (b) promoting trade in goods, services and investment capital [54]. However, other researchers have also found that without clear political priorities, policy-making is also hampered by lack of clear direction and purpose [35, 36, 39, 55]. Examining how the MOA and the MoHMS access, apply and share evidence is therefore important for better understanding of their existing structures and functions towards improved collaboration.

As noted, the findings from this study demonstrate the absence of a clear formal direction that guides effective communication between potential partners. Efforts are needed for the MoHMS and the MOA to harmonize the policy approaches and methods, at least within areas with similar policy interests. Cross-sectoral collaboration that involves linking or sharing of evidence, policies, resources, activities and services by two or more organizations in the pursuit of a common goal [56] is one of the successful strategies [57]. However, challenges around building trust and coordinated responses were reported. At the outset, stakeholders need to know exactly what they had to offer and to understand the problem well enough to know how they might be interdependent. The barriers identified in this current study and in line with findings by Thow and others [39, 58], indicate areas that government organizations can target to build a system that supports collaboration in policy-making.

### Competence

The other most essential element of using evidence as highlighted by participants was competence in collecting, analyzing and critically appraising evidence. Effective use of evidence is determined by the policy-maker’s ability to access and analyse the best available evidence, and apply it to the formulation of policies. However, strengthening capacity building and providing incentives are key to the successful adoption of and support for the development of evidence-informed policies [20–22].

Many participants recognized the value and importance of using evidence in policy-making but did not have either the time or skills to search for the evidence themselves. Various strategies have been used to promote policy dialogues [48] like knowledge brokerage mechanisms [28, 29, 59] and presenting research results to policy-makers [60]; nevertheless, the uptake of evidence still remains an important challenge to policy-making. While training all staff in an organization on the use of evidence in policy-making seems necessary, it is not sufficient to support evidence-informed policy; researchers are searching for more effective and innovative mechanisms to bridge the gap between knowledge generation and uptake [61]. Bunger et. al observed that engaging a trained group of staff as experts who will act as information sources and champions to whom policy-makers can turn when they need knowledge and evidence would provide for improved evidence-uptake in a more centralized network [62]. Pursuing this approach in Fiji should help the existing landscape of information providers and their information systems technologies and databases. This may help to overcome at least some of the difficulties faced by policy-makers when trying to bring together information from different sources and institutions on the same subject or area of interest.

### Strengths and limitations of the study

The strengths of this study are that we were able to conduct face-to-face interviews with actual policy-makers; the involvement and cooperation of two government ministries; and the willingness of participants to engage and focus on authentic policy processes. A limitation was that, even though we assured interviewees that the data would be anonymized, some may have held back information through fear of repercussions. Also, not all key documents such as discussion papers and cabinet papers could be accessed; these may have been useful in developing a better understanding of the interview data and the wider context.

## Conclusions

This study has shown that evidence is sometimes used in the process of making food-related policies in Fiji but research tools, technical knowledge to access and use sound and relevant evidence, and collaboration with other government sectors stand in the way of more consistent and informed use of evidence. Each Ministry’s Executive Committee members required evidence to support policy briefs/proposal. Where insufficient or poor quality evidence was provided, this had the effect of slowing down the approval process because the Executive Committee would send the brief back for further input. In some cases evidence simply wasn’t available and this could stall policy implementation indefinitely.

### Implications

There is a need for an active network and functional information system in the Ministries comprising context-specific knowledge management and translation opportunities linking people and resources and building on existing expertise and professional networks. This provides a broad integrated view of dynamics associated with the use of evidence in the process of food-policies, thus accommodating the different perspectives of policy-makers. This study has shown that organizations need to look at all aspects of their systems to ensure that at every level and point in the process, evidence access and use is supported and encouraged.

## Abbreviations

C-POND: Pacific Research Centre for the Prevention of Obesity and Non-communicable Diseases
MOA: Ministry of Agriculture
MoHMS: Ministry of Health and Medical Services
NCDs: Non-communicable Diseases
OPIC: Pacific Obesity Prevention In Communities
TROPIC: Translational Research On Obesity Prevention In Communities
WHO: World Health Organisation
